# A Randomised Controlled Trial of a Brief Online Mindfulness-Based Intervention on Paranoia in a Non-Clinical Sample

**DOI:** 10.1007/s12671-017-0774-2

**Published:** 2017-07-14

**Authors:** Robert Shore, Clara Strauss, Kate Cavanagh, Mark Hayward, Lyn Ellett

**Affiliations:** 1 School of Psychology, University of Surrey, Guildford, Surrey, GU2 7XH UK; 2 School of Psychology, University of Sussex, Falmer, East Sussex, BN1 9RH UK; 3 Sussex Partnership NHS Foundation Trust, Mill View Hospital, Nevill Avenue, Hove, BN3 7HY UK; 4 Department of Psychology, Royal Holloway, University of London, Egham, Surrey TW20 0EX UK

**Keywords:** Mindfulness, Paranoia, RCT, Mediation, Online

## Abstract

Paranoia is common and distressing in the general population and can impact on health, emotional well-being and social functioning, such that effective interventions are needed. Brief online mindfulness-based interventions (MBIs) have been shown to reduce symptoms of anxiety and depression in non-clinical samples; however, at present, there is no research investigating whether they can reduce paranoia. The current study explored whether a brief online MBI increased levels of mindfulness and reduced levels of paranoia in a non-clinical population. The mediating effect of mindfulness on any changes in paranoia was also investigated. One hundred and ten participants were randomly allocated to either a 2-week online MBI including 10 min of daily guided mindfulness practice or to a waitlist control condition. Measures of mindfulness and paranoia were administered at baseline, post-intervention and 1-week follow-up. Participants in the MBI group displayed significantly greater reductions in paranoia compared to the waitlist control group. Mediation analysis demonstrated that change in mindfulness skills (specifically the observe, describe and non-react facets of the FFMQ) mediated the relationship between intervention type and change in levels of paranoia. This study provides evidence that a brief online MBI can significantly reduce levels of paranoia in a non-clinical population. Furthermore, increases in mindfulness skills from this brief online MBI can mediate reductions in non-clinical paranoia. The limitations of the study are discussed.

## Introduction

Paranoid thinking occurs when individuals believe that harm is occurring or going to occur and that others intend to cause harm (Freeman and Garety 2000). Although paranoid beliefs traditionally are associated with clinical diagnoses such as schizophrenia, recent research (e.g. Ellett et al. 2003; Freeman et al. 2011; Freeman and Garety 2014; Johns et al. 2004) supports the postulate that paranoid thinking is also common in the general population (Fenigstein and Vanable 1992). Indeed, recent research suggests that different types of paranoid ideation occur in up to 30% of the general population (Bebbington et al. 2013). This is consistent with a dimensional understanding of mental health, within which an experience such as clinical paranoia is held to lie on continuum with paranoia seen in the general population (Strauss 1969). From an evolutionary perspective, it has been proposed that paranoia is a trait that was selected and distributed in humans due to its adaptive value in past ancestral environments (Ellett et al. 2003). Studies have also shown that paranoia can be both distressing and preoccupying in the non-clinical population (Freeman et al. 2011; Ellett et al. 2003), is persistent (Allen-Crooks and Ellett 2014), slow to dissipate once activated (Ellett and Chadwick 2007) and associated with anxiety and depression (Freeman et al. 2013). Therefore, it is important that effective interventions are available for people experiencing distress from paranoia across the continuum of experience. Distress from paranoia can be difficult to reduce using a cognitive reappraisal approach (Chadwick 2006; Ellett 2013), and mindfulness offers the opportunity to potentially reduce distress without directly challenging the content of beliefs.

Mindfulness is characterised by an intentional, non-judgemental awareness of present-moment experiences such as thoughts, feelings, sounds and physical sensations (Kabat-Zinn 2000). Mindfulness-based interventions (MBIs) are typically 8-week group interventions that teach people mindfulness skills through in-session and home-based practice, combined with discussion about what was learned from the practice. A recent meta-analysis including 209 studies examined the effectiveness of MBIs with clinical and non-clinical populations (Khoury et al. 2013). Waitlist controlled studies targeting psychological disorders found medium to large between-group effect sizes for both anxiety (*n =* 4; *g* = 0.96; 95% CI [0.67, 1.24]) and depression (*n =* 8; g = .53 (95% CI [0.32, 0.73]). Studies using another psychological treatment as a control showed a small between-group effect size in favour of MBIs (*g* = 0.22; 95% CI [0.12, 0.33]), with MBIs producing comparable effect sizes to traditional CBT or behaviour therapies *(n =* 9; *g =* −0.07; 95% CI [−0.26, 0.16]).

Although MBIs are often delivered using an 8-week protocol of weekly 2-h sessions, as is the case in mindfulness-based stress reduction (MBSR; Kabat-Zinn 1990) and mindfulness-based cognitive therapy (MBCT; Segal et al. 2002), recent research has begun to examine the effectiveness of using self-help methods such as books or online courses (Cavanagh et al. 2014). Indeed, the time and resource intensive nature of MBIs can be prohibitive to potential participants and service providers (Crane and Kuyken 2013) and the accessibility of MBIs could be substantially increased through self-help resources such as books, audio recordings, online courses or smartphone apps. A meta-analysis of randomised controlled trials of self-help mindfulness and acceptance-based interventions with both clinical and non-clinical populations found effects relative to control conditions on measures of mindfulness (*g =* 0.49; 95% CI [0.23, 0.76]), depression (*g =* 0.37; 95% CI [0.18, 0.56]) and anxiety (*g =* 0.34; 95% CI [0.10, 0.56]) (Cavanagh et al. 2014). To date, there have been relatively few studies using brief MBIs of fewer than four sessions; however, those that have been conducted have shown promising results for the effectiveness of brief MBIs on a range of outcome measures (Zeidan et al. 2010; Call et al. 2014). A study by Cavanagh et al. (2013) that used the same brief online intervention as the current study in comparison to a waitlist control invited participants (students) to practice mindfulness daily for 10 min using a 2-week online MBI course. On measures of mindfulness, perceived stress, anxiety and depression, medium post-intervention between-group effect sizes were found, ranging from *d =* 0.41 to *d =* 0.62. This indicates how a brief MBI of 2 weeks in duration may be effective at reducing distress in a non-clinical population.

Whilst most mental health MBI research has focused on the effectiveness of MBIs for depression and anxiety, quantitative and qualitative research has begun to explore the potential of MBIs for psychosis (Chadwick et al. 2005; Abba et al. 2008; Strauss et al. 2015). A recent randomised controlled trial found mindfulness to enhance psychological quality of life in patients with a diagnosis of schizophrenia spectrum disorders (Lopez-Navarro et al. 2015). Adopting a single-symptom approach to psychosis, research has also indicated potential benefits of mindfulness in more than 60 people with distressing psychotic voices (Dannahy et al. 2011; Goodliffe et al. 2010; May et al. 2014; Chadwick et al. 2009)—but to date, there are only two single cases evaluating MBIs for clinical paranoia (Ellett 2013) and one randomised controlled trial comparing MBCT with a waitlist control on daily life ratings of paranoia in a sample of individuals with a history of at least one episode of major depressive disorder (Collip et al. 2013).

There are compelling reasons to further develop and test MBI interventions for paranoia. First, a recent meta-analysis of randomised controlled trials of cognitive behaviour therapy (CBT) for delusions (including paranoia) found only a small effect size (delusions, *g* = 0.24; van der Gaag et al. 2014). Second, paranoia can be particularly difficult to treat using traditional cognitive reappraisal methods (Chadwick 2006; Ellett 2013). An MBI approach seeks to reduce distress without directly challenging the content of beliefs (Vilardaga et al. 2013), focussing instead on letting go of reactions such as self-judgement and rumination on paranoid thoughts, as well as acceptance of self and (psychotic) experience (Abba et al. 2008; Chadwick et al. 2009). Additional research using methodologically robust designs examining the effectiveness of MBIs on paranoia is now warranted.

The current study used a waiting list RCT design to evaluate the effects of a brief online MBI on paranoia in a non-clinical population. Repeated measures were taken at baseline, after the 2-week intervention and at 1-week follow-up for each condition. The study tested the following hypotheses: (1) participation in an online MBI will lead to greater reductions in paranoia at post-intervention and follow-up compared to a waitlist control, and (2) improvements in mindfulness will mediate the relationship between intervention type (MBI or wait-list) and reductions in paranoia.

## Method

### Participants

Previous research using a matched online MBI found medium effect sizes ranging from *d* = 0.41 to *d* = 0.62 on measures of mindfulness, stress, anxiety and depression (Cavanagh et al. 2013) in a non-clinical sample. Taking this into account, medium effect sizes were assumed, and therefore, to test the mediation model with 80% power (*p* = .05) in a bias-corrected bootstrapped mediation analysis, a minimum of 36 study completers per condition were needed (Fritz and MacKinnon 2007). Inclusion criteria were that participants had to be over 18 years of age, able to understand English and have capacity to consent to the study. There were no exclusion criteria.

Participants were 110 people (48% were students and 89% were female) who were recruited either through a British university or via posts on social media websites. The sample age ranged from 18 to 67 years old (*M* = 32.16 years, *SD* = 13.57 years). The study protocol was approved by the host University Ethics Committee, and all participants gave online informed consent prior to participation.

### Procedure

Following informed consent, all participants completed baseline questionnaires online. Within 7 days of completing baseline questionnaires, participants were randomised, using a computer-generated block random allocation method, to either the mindfulness-based intervention (immediate start) or waitlist control condition. The intervention was accessible via a hyperlink supplied to participants. Participants completed the questionnaires (FFMQ and PS only) at the end of the intervention (i.e. after 2 weeks) and again at follow up (i.e. 1 week after the intervention had ended). Individuals in the mindfulness group also completed the participant engagement questionnaire at two points in the study—at the end of the first week (to assess level of engagement and to remind them to continue accessing the material) and at the end of the second week. Individuals in the waitlist group were given access to the online intervention at the end of the study once follow-up measures were completed.

#### Online Mindfulness-Based Intervention

The ‘Learning Meditation Online’ intervention was the intervention used by Cavanagh et al. (2013) with some minor adaptations. It was a webpage hosted by the University of Surrey using Sawtooth Software technology (Sawtooth Software Inc. SSI WEB program v8.3, Sequim, Washington 2013). The website was divided into six sections. The first section ‘what is mindfulness?’ contained information about the purpose and benefits of learning mindfulness with a 5-min video clip introducing the concept of mindfulness. The second section ‘daily mindfulness practice’ provided a 10-min guided mindfulness meditation in a male and female voice that was recorded from a script developed by Chadwick (2006), and comprised a brief body scan, followed by mindful breathing and choiceless awareness. The people who recorded the guided mindfulness meditation were experienced in practicing and delivering MBIs. Section three ‘everyday mindfulness activities’ provided information on how to bring mindfulness to everyday activities. The fourth section had frequently asked questions with answers to provide information about what to expect when practicing mindfulness. Section five contained information about the study and section six gave contact details for the research team alongside help and assistance such as counselling services and mental health charities. The webpage was self-guided and e-mail addresses of the research team were only provided in the case of technical difficulties.

### Measures

#### Five-Facet Mindfulness Questionnaire (Baer et al. 2006)

The Five-Facet Mindfulness Questionnaire (FFMQ) is a self-report scale that is used to measure how mindful individuals are in their daily lives. It has 39 items with each item rated on a 5-point Likert-type scale from 1 ‘never or rarely true’ to 5 ‘very often or always true’, and total scores for the FFMQ range between 39 and 195. It identifies five independent facets of mindfulness and therefore allows investigation into which aspects of mindfulness might be mediating change. The independent facets are observing (8 items; range 8–40), describing (8 items; range 8–40), acting with awareness (8 items; range 8–40), non-judging of inner experience (8 items; range 8–40) and non-reactivity to inner experience (7 items; range 7–35). At present, it is the most commonly used mindfulness questionnaire and it is based on a factor analysis of items from five frequently used mindfulness questionnaires (De Bruin et al. 2012). The scale has demonstrated adequate to good internal consistency for all five facets (α = 0.75–0.91), and the five facets of mindfulness have been shown to be robust for different types of samples including meditators, non-meditators, students and the general population (Baer et al. 2006; Baer et al. 2008). Cronbach’s alpha for the full FFMQ at baseline in the current study was 0.94.

#### Paranoia Scale (Fenigstein and Vanable 1992)

The Paranoia Scale (PS) is a 20-item self-report scale. Each item is rated on a 5-point Likert-type scale from 1 ‘not at all applicable’ to 5 ‘extremely applicable’ with a range of total scores between 20 and 100. The PS was developed to measure paranoia in college students and is not a clinical tool for diagnosing clinical paranoia, and therefore, there are no specific cut-off scores to indicate severity of paranoia. In a sample of college students, the mean total score on the paranoia scale was 42.7 (SD = 10.2; Fenigstein and Vanable 1992). It is the most widely used measure of paranoia, and it has demonstrated good internal and test re-test reliability (α = 0.84, *r* = 0.70; Fenigstein and Vanable 1992). Cronbach’s alpha for the scale at baseline in the current study was 0.92.

#### Participant Engagement Questionnaire

This questionnaire was adapted from Cavanagh et al. (2013) and assessed participant engagement with the mindfulness-based intervention over the previous week using five questions. Four questions asked about the amount of time (free text) and the number of days (0–7) participants spent engaging in course materials, listening to the audio meditation and engaging in meditation practice. In order to assess participants’ experience of the intervention, the final question used a Likert scale to enquire how much participants felt the intervention was improving their wellbeing (*1 = not at all* to *9 = very much*). The number of times the website was accessed by each participant was also recorded. In the current study, Cronbach’s alpha for the four items was 0.75 and test-retest reliability for the scale was 0.60.

### Data Analyses

Skew and kurtosis were calculated for each variable and were found to be within acceptable bounds (i.e. <2.58). There was also some missing data for both the paranoia (*n* = 38 at Time 2 and 3) and mindfulness (*n* = 40 at Time 2 and 3) measures. Comparisons were made of means and standard deviations at baseline to check for differences between groups prior to randomisation in order to check group equivalence. All data were checked to ensure assumptions for multiple regression were met. Residuals from each path of the mediation model (see Fig. 2) were checked for normality of distribution, homoscedasticity and independent errors. Predictor variables were checked for zero variance and multicollinearity. Outcome variables were checked for independence and linearity and outliers were checked using Cook’s distances. Scatterplots showed linearity between variables, histograms of the residuals for each pathway showed they were normally distributed and Cook’s Distance tests were all less than 1.1 indicating no overly influential outliers. Therefore, the assumptions necessary for bootstrapped mediation analysis were satisfactorily met.

To test hypothesis one, a mixed ANOVA was conducted with post hoc tests where warranted exploring the effects of group (MBI or waitlist) and time (pre-intervention and follow-up) on paranoia scores. For the mediation analysis, standardised residual change scores were calculated for both mindfulness skills pre-post intervention and for paranoia scores pre-1 week post-intervention, which allowed for changes in the mediator to be measured prior to changes in paranoia. The mediation analysis was conducted using the PROCESS macro for SPSS (Hayes 2013) using 5000 resamples and bias corrected and accelerated confidence intervals (BCa CI). This gives total and indirect effects with both bootstrapping confidence intervals and the product-of-coefficients approach. Bootstrapped 95% confidence intervals for the a × b effect were used. This approach is more powerful than the Baron and Kenny (1986) causal steps approach with a lower risk of type II errors. It is also more robust in the event that multiple regression assumptions are violated, and it does not incorrectly assume that path c needs to be significant in order for mediation to occur (Hayes 2008). Unstandardised beta (β) coefficients for the pathways on the mediation model are also reported.

## Results

One hundred and ten participants were randomly allocated to either the online mindfulness-based intervention or to the waitlist control condition. The mean age for the MBI group was 32.5 years (SD = 13.5) and for the waitlist was 31.9 years (SD = 13.8). The majority of participants were female in both the MBI (83.9%) and the waitlist group (94.4%). Sample characteristics for each condition at baseline are displayed in Table 1. No significant between-group (MBI vs waitlist) differences were found at baseline in terms of age, gender and level of education, or on the Paranoia Scale (*t*(108) = −0.35, *p* = .73) or FFMQ (*t*(108) = 0.09, *p* = .37). Furthermore, there were also no significant gender differences at baseline on either paranoia (*t(*108) = 1.19, *p* = .24) or mindfulness (*t(*108) = .60, *p* = .55). Pre, post and follow-up data for the Paranoia Scale and the FFMQ are displayed in Table 2.

**Table 1:** Characteristics of the MBI and waitlist groups at baseline

Variable	MBI (*N* = 56)	Waitlist (*N* = 54)	Statistics
Mean age (years) (SD)	32.5 (13.5)	31.9 (13.8)	*t*(108) = −0.24, *p* = .81
Gender—% female	83.9%	94.4%	χ^2^(1) = 3.13, *p* = .08
Ethnicity—% White British	62.5%	64.8%	FET = 8.32, *p* = .36
% live in UK	83.3%	86.5%	χ^2^(1) = 0.21, *p* = .65
% ‘A’ levels as highest level of education	43.4%	42.5%	FET = 6.00, *p* = .56

**Table 2:** Descriptive statistics on study variables measures at all time points

Variable	MBI Mean (SD)			Waitlist Mean (SD)		
	Pre	Post	Follow-up	Pre	Post	Follow-up
Paranoia Scale^a^	39.1 (13.1)	31.0 (10.8)	29.3 (10.5)	41.1 (13.6)	40.4 (13.9)	36.6 (12.8)
FFMQ observing	24.6 (6.4)	27.6 (4.7)	29.3 (5.8)	23.9 (5.5)	23.0 (5.3)	23.3 (6.6)
FFMQ describing	26.3 (7.2)	27.7 (5.5)	28.3 (6.6)	26.1 (6.9)	24.2 (6.6)	25.3 (6.9)
FFMQ act with awareness	22.2 (5.9)	25.5 (4.5)	25.9 (4.2)	22.4 (6.5)	22.2 (6.3)	23.1 (6.6)
FFMQ non-judging	24.1 (6.6)	26.4 (5.7)	28.2 (6.3)	24.2 (6.9)	25.4 (3.9)	26.3 (7.3)
FFMQ non-reacting	17.8 (4.9)	20.7 (4.3)	21.0 (5.2)	18.9 (4.7)	18.8 (3.9)	19.1 (4.5)

^a^Possible range of scores 20–100; negative changes are improvements; published mean in non-clinical sample 42.7 (Fenigstein and Vanable 1992)

**Fig. 1:**
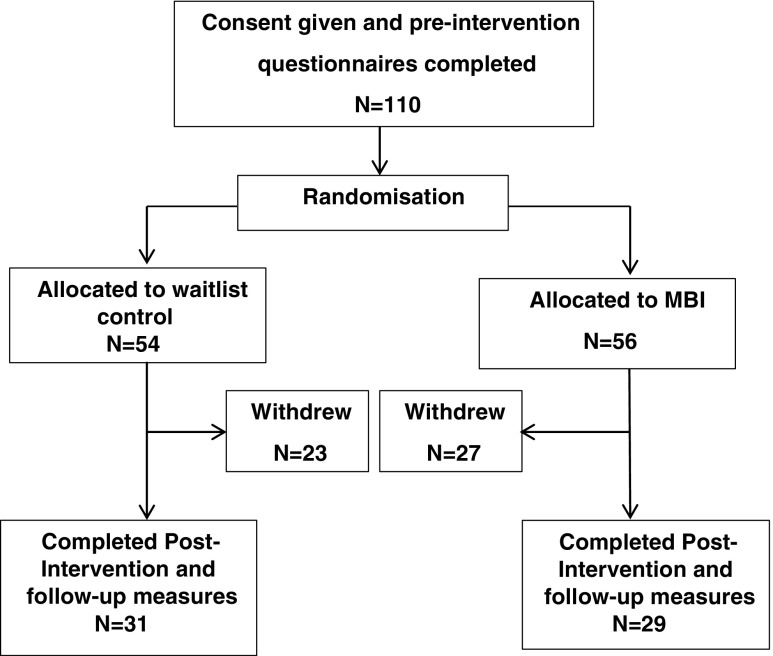
Consort diagram outlining the participant flow through the study

Data analysis was conducted on participants who completed measures at all three time points. Of the 110 participants, 60 (55%; MBI = 29, waitlist = 31) provided complete data and were included in the analysis. This did not meet the sample size identified in the a priori power analysis; however, mediation effect sizes were typically large and this suggests that the a priori power calculation was overly conservative. Therefore, the smaller sample size of 60 was adequate to detect a large mediation effect on most of the FFMQ subscales. A consort diagram outlining the participant flow through the study is shown in Fig. 1. There was no difference in completion rates between the groups; however, this finding did approach significance, *t*(106) = −1.80, *p* = .07. Furthermore, no significant differences were found between participants who completed and those that dropped out with respect to age (*t*(83) = 0.24, *p* = .81), gender (*t*(41) = 1.51, *p* = .14) or baseline scores on the Paranoia Scale (*t*(108) = −0.93, *p* = .35) or FFMQ (*t*(108) = −0.74, *p* = .46).

There was a significant group by time interaction on paranoia (*F*(1.70, 98.72) = 5.70, *p* = .01). Between-group *t* tests showed a significant difference between MBI and waitlist control at post-intervention (*t*(69.9) = 2.32, *p* = .024, *d* = 0.74 95% CI for *d =* (0.22, 1.27)) and at follow-up (*t*(66) = 2.364, *p* = .021, *d* = 0.62, 95% CI for *d =* (0.12, 1.10)). Within-group *t* tests indicated that the MBI group showed a significant decrease in paranoia over time both pre- to post-intervention (*t*(33) = 4.18, *p* < .001, *d* = 0.60, 95% CI for *d =* (0.11, 1.08)), and pre-intervention to follow-up (*t*(30) = 5.34, *p* < .001, *d* = 0.80, 95% CI for *d =* (0.27, 1.30)). Within-group *t* tests for the waitlist control group showed no significant change in paranoia pre- to post-intervention (*t*(37) = 0.07, *p* = .95, *d* = 0.01, 95% CI for *d =* (−0.44, 0.46)). However there was a significant decrease in paranoia pre-intervention to follow-up in the waitlist control group with a small effect size (*t*(36) = 2.72, *p* = .01, *d* = 0.29, 95% CI for *d =* (−0.18, 0.74)).

**Fig. 2:**
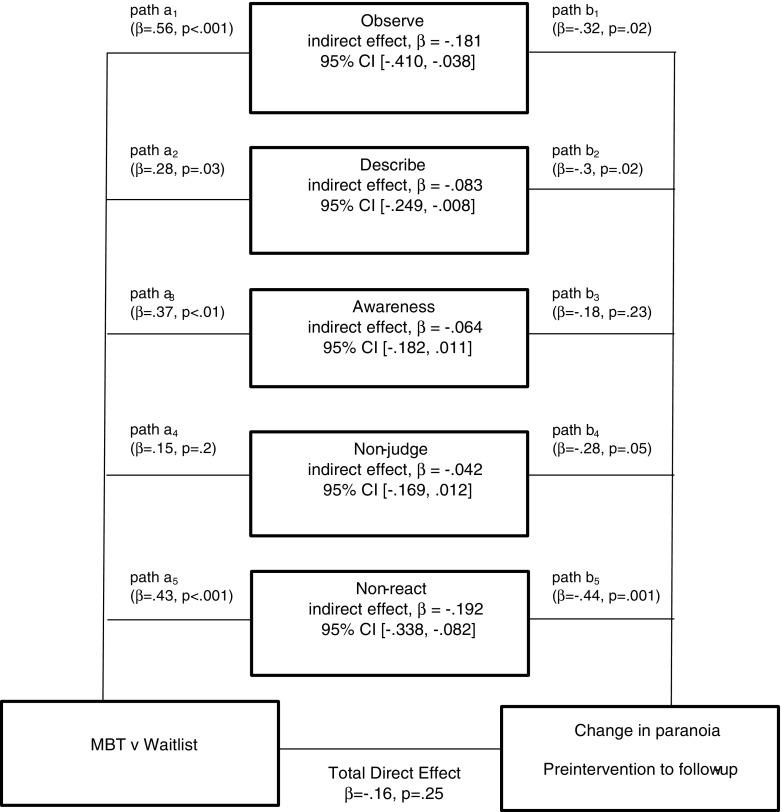
Coefficients for the pathways showing the mediational effect of change in mindfulness score post-intervention on paranoia score at follow-up adjusted for baseline values

A summary of the mediation analysis is presented in Table 3. There was a significant indirect effect of treatment condition on paranoia change through change in the observe subscale (β = −0.18, 95% BCa CI −0.41, −0.04), the describe subscale (β = −0.08, 95% BCa CI −0.25, −0.01) and the non-react subscale (β = −0.19, 95% BCa CI −0.34, −0.08). However, there was no significant indirect effect through change in the Awareness subscale (β = −0.06, 95% BCa CI (−0.18, 0.01) and the non-judging subscale (β = −.04, 95% BCa CI (−0.17, 0.01). The unstandardised beta coefficients for the pathways on the mediation model are illustrated in Fig. 2. This shows there was no significant direct effect for treatment condition on paranoia change β = 0.16, *p* = .25.

**Table 3:** Summary of mediation analysis showing the mediational effect of change in mindfulness score post-intervention on paranoia score at follow-up, adjusted for baseline values

Independent variable	Mediating variable	Dependent variable	Effect of IV on DV	Effect of M on DV	Direct Effect	Indirect effect	
(IV)	(M)	(DV)	(a)	(b)	(c’)	(a × b)	95% CI
Group	Observe	Paranoia change	0.56	−0.32	−0.19^a^	−0.18	(−0.41, −0.04)
	Describe		0.28	−0.30	−0.28	−0.08	(−0.25, −0.01)
	Aware		0.37	−0.18	−0.30	−0.06^a^	(−0.18, 0.01)
	Non-judge		0.15^a^	−0.28	−0.32	−0.04^a^	(−0.17, 0.01)
	Non-react		0.43	−0.44	−0.17^a^	−0.19	(−0.34, −0.08)

^a^Non-significant results at 95% confidence

Data regarding the level of engagement with the MBI was obtained from the Participant Engagement Questionnaire (PEQ) after 1 week and at post-intervention. Twenty-four participants (83%) completed the PEQ, and the mean number of self-reported days of practice was 11.83 days (range 5–16 days, SD = 3.68). Mean reported scores from the PEQ for level of well-being were 5.03 (week 1) and 5.23 (week 2).

## Discussion

Our study shows that a brief online MBI can reduce levels of paranoia in a non-clinical population and demonstrated that this effect was mediated by an increase in mindfulness skills, specifically the Observe, Describe and Nonreact subscales of the FFMQ. The findings from the current study support those of Ellett (2013), who used an individual six session MBI for two people experiencing persecutory delusions without distressing voices, and Collip et al. (2013) who found that MBCT (compared to waitlist control) reduced daily life ratings of paranoia in individuals with a history of major depressive disorder. The findings extend the current evidence base of MBIs for paranoia using a more robust RCT design. The findings are also consistent with research evidence indicating that change in mindfulness mediates the relationship between MBIs and improved psychological wellbeing across a range of difficulties, including perceived stress, positive states of mind, post-traumatic avoidance symptoms, depressive symptoms and general psychological functioning (Baer 2009; Bränström et al. 2010; Gu et al. 2015; Kuyken et al. 2010).

The findings from this study compliment the findings of Cavanagh et al. (2013), who reported moderate between-group effect sizes on measures of mindfulness, perceived stress, anxiety and depression in a non-clinical sample. Taken together, these studies extend the emerging evidence base for the effectiveness of online self-help MBIs and suggest that a brief 2-week online MBI can improve levels of mindfulness and effectively reduce a range of psychological symptoms in a non-clinical population. The findings also contribute to the ongoing debate in the literature regarding the frequency and duration of mindfulness practice needed to facilitate change. In both the current study and Cavanagh et al. (2013), engaging in mindfulness practice for just 10 min a day over a 2-week period was sufficient to facilitate change. This provides some evidence for recent suggestions in the literature that briefer MBIs with shorter practices can be effective in reducing symptoms of psychological distress and negative mood in non-clinical populations (Virgili 2013; Zeidan et al. 2010), pain-related distress in those with chronic pain (Ussher et al. 2014) as well as for those from clinical populations experiencing a current episode of major depressive disorder (Strauss et al. 2014). This is an important finding because it demonstrates the effectiveness of brief online MBIs that have relatively low financial and resource costs to providers. It will be important for future research to demonstrate whether these effects can be extended to people from clinical populations experiencing clinically significant paranoia and persecutory delusions. This is particularly important given the recent calls in the literature to markedly improve the effectiveness of psychological interventions for delusional (including paranoid) beliefs (van der Gaag et al. 2014; Freeman and Garety 2014; Chadwick et al. 2016). Mindfulness-based interventions offer a particularly promising alternative approach given that they have the potential benefit of reducing distress related to paranoia, without directly challenging the content of beliefs (Chadwick 2006; Ellett 2013).

### Limitations

There are a number of limitations of the study that warrant consideration. Despite significant indirect effects from three subscales of the FFMQ, the mediation analysis did not find a significant indirect effect from the awareness and non-judging subscales of the FFMQ. The 95% confidence intervals in these two cases only just cross 0 which indicates that the study may have been underpowered to find significant indirect effects for these subscales. Therefore, it would be beneficial for the study to be replicated with a sample size large enough to detect potential indirect effects in the awareness and non-judging subscales. An active control condition was not used in this study, and therefore, it remains unclear whether the changes observed are due to mindfulness practice or other non-specific factors such as expectation of benefit. Furthermore, use of a waitlist control meant that it was not possible to demonstrate whether the MBI was more effective than other interventions, such as online CBT.

Participants were not screened for any previous or current meditation practice or involvement in other psychological interventions; therefore, the changes found could have occurred due to participants engaging in additional meditation practice or other psychological interventions outside of the online intervention. Additionally, we did not use a diagnostic screening tool, and the paranoia measure used in the study does not have severity cut-offs; therefore, it was not possible to determine whether any participants were experiencing clinically significant symptoms. The results indicated a small but significant decrease in paranoia from baseline to follow-up in the waitlist control group. This could be due to regression to the mean or may represent an expectancy effect in the responses of participants.

The level of attrition from the study had the potential of causing some bias in the data. However, there were no significant differences between participants who completed and those that dropped out with respect to age, gender and baseline scores on the paranoia scale or FFMQ. Attrition rates were also comparable to those reported in other online intervention studies (Melville et al. 2010). Although the gender ratio was skewed with the majority of participants being female, which could impact on the generalisability of our findings, there were no significant gender differences in the two study groups (MBI vs waitlist) or on any of the study variables at baseline. As is the case in other online mindfulness studies (e.g. Cavanagh et al. 2013), we used a retrospective self-report measure of mindfulness practice, which may be subject to a range of biases, such as memory and mood biases.

Although follow-up data were collected, the follow-up period itself was only 1 week, which limits the conclusions that can be made with regard to whether changes in both paranoia and mindfulness are maintained over a longer period. Future research might usefully examine the extent to which changes can be maintained over a longer follow up period, whether individuals continue to engage in mindfulness practice after formal participation in the study has ceased, and the extent to which ongoing practice has a cumulative effect on reductions in paranoia. We only considered one potential mediating factor (mindfulness skills) and did not measure any other factors that are known to be important in the onset and maintenance of paranoid cognitions, such as negative schematic beliefs, worry and rumination (Freeman et al. 2013; Chadwick et al. 2009; Paget and Ellett 2014). Future research should therefore examine the extent to which mindfulness-based interventions impact on these important cognitive and emotional processes. Finally, the study employed a non-clinical sample; therefore, further research is needed to determine whether our findings generalise to individuals with clinical paranoia.
